# Insights into the production of erythritol by strains of *Limosilactobacillus fermentum* of sourdough origin

**DOI:** 10.1128/aem.01565-25

**Published:** 2025-09-24

**Authors:** Víctor González-Alonso, Marko Verce, Inés Pradal, Frédéric Leroy, Luc De Vuyst

**Affiliations:** 1 Research group of Industrial Microbiology and Food Biotechnology (IMDO), Faculty of Sciences and Bioengineering Sciences, Vrije Universiteit Brussel 70493, Brussels, Belgium; The Pennsylvania State University, University Park, Pennsylvania, USA

**Keywords:** sourdough, erythritol, sugar alcohol, lactic acid bacteria

## Abstract

Erythritol is a sugar alcohol that is occasionally produced by heterofermentative lactic acid bacteria (LAB). Although such biosynthesis has rarely been reported during sourdough production, the potential of the sourdough strains *Limosilactobacillus fermentum* IMDO 130101 and IMDO TC9L10 to produce erythritol was demonstrated in the context of their broader metabolic capacity both during wheat sourdough productions and in a wheat sourdough simulation medium (WSSM). Indeed, both strains produced up to 0.4 mM of erythritol during starter culture-initiated wheat sourdough productions. The strain *L. fermentum* IMDO 130101 was further used to carry out controlled fermentation processes in WSSM and was able to synthesize 0.53 ± 0.03 mM of erythritol. During both the wheat sourdough productions and WSSM fermentation processes, acetate was co-produced with erythritol and/or glycerol. The primary role of erythritol was likely its involvement in redox balancing. Besides, the pathways for erythritol production were probed *in silico*, but no genes corresponding with erythrose-producing or erythrose-converting enzymes could be identified. Therefore, it was suggested that the enzymes responsible for the production of erythritol in *L. fermentum* IMDO 130101, and other heterofermentative LAB species, are the same as those producing mannitol or glycerol.

**IMPORTANCE:**

Lactic acid bacteria have a limited biosynthesis capacity. Their main carbohydrate breakdown pathways enable them to produce energy and to maintain their redox balance. The latter is accomplished through the production of lactic acid in the case of homofermentative lactic acid bacteria and ethanol or acetic acid in the case of heterofermentative ones. However, under certain conditions, other branches of this pathway become active, which lead to end-metabolites that are produced seldomly. An example of such an end-metabolite is erythritol, which was already detected during sourdough production but never investigated in detail. The present study showed the production of erythritol by *Limosilactobacillus fermentum* inhabiting sourdough environments and its involvement in redox balancing.

## INTRODUCTION

Erythritol is a sugar alcohol (polyol), a low-digestible compound obtained by the reduction of erythrose (1). Sugar alcohols can be produced by plant, fungal, and bacterial cells for several reasons, i.e., as carbon storage molecules, for redox balancing, or to protect against osmotic and oxidative stresses (2–4).

Lactic acid bacteria (LAB) are sugar alcohol producers thanks to their versatile and efficient carbohydrate conversion and branching pathways (5, 6). Previous research on sugar alcohol production by LAB has focused mostly on glycerol, mannitol, and sorbitol. First, mannitol production by heterofermentative LAB species is generally well characterized (7, 8). To do so, fructose is internalized into the bacterial cell and reduced to mannitol by a mannitol dehydrogenase, as a way to recuperate NAD^+^ and, hence, produce acetate and extra ATP instead of ethanol for redox balancing (without ATP production) to improve competitiveness. Mannitol dehydrogenase typically favors NADH over NADPH as cofactor (9). This mechanism has been described for LAB species, such as *Fructilactobacillus sanfranciscensis*, *Limosilactobacillus pontis*, *Limosilactobacillus fermentum*, and *Leuconostoc citreum* (10, 11). Second, glycerol is produced by heterofermentative LAB species, such as *L. citreum* and *Oenococcus oeni*, starting from glyceraldehyde 3-phosphate and depending on glycerol 3-phosphate dehydrogenase activity to form glycerol 3-phosphate, followed by dephosphorylation of the latter through phosphatase activity (12). Third, sorbitol does not seem to be naturally produced by LAB, but its production has been achieved via genetic engineering of *Lacticaseibacillus casei* (13, 14). Finally, the production of arabitol, ribitol, and xylitol by LAB has also been explored through metabolic engineering, by acting on the pentose phosphate pathway (PPP) at the stage of either ribulose 5-phosphate (arabitol and ribitol) or xylulose 5-phosphate (arabitol, ribitol, and xylitol) (14). Xylitol can also be produced by an engineered strain of *Lactococcus lactis* NZ9800 that encodes a xylose reductase from yeast origin, which reduces D-xylose to xylitol (15).

In contrast to mannitol and glycerol, which are mainly produced as osmolytes, the production of erythritol by LAB has seldom been reported. Exceptions are a low erythritol-producing activity by some strains of *O. oeni* (16, 17) as well as *F. sanfranciscensis* (7, 11) and *Fructilactobacillus florum* (18). *Fructilactobacillus sanfranciscensis* is a typical sourdough LAB species (19, 20). It is likely that certain sourdough matrix conditions promote erythritol biosynthesis to favor NAD(P)H redox balancing. Indeed, during sourdough production, an accumulation of up to 0.14 g/kg of erythritol in wheat and spelt sourdoughs has been recorded, possibly elaborated by *L. fermentum*, *Lactiplantibacillus plantarum*, or *L. citreum*, although it was not certain if these species were responsible for the erythritol production (21). Similarly, production of erythritol has been linked to *L. fermentum* during backslopped sourdough productions carried out with triticale flour (22) and starter culture-initiated sourdough productions with wholemeal wheat flour (23). In addition to erythritol, the heterofermentative carbohydrate breakdown by *L. fermentum* IMDO 130101 yields lactic acid, ethanol, mannitol, glycerol, and acetic acid (22–24).

**Fig 1:**
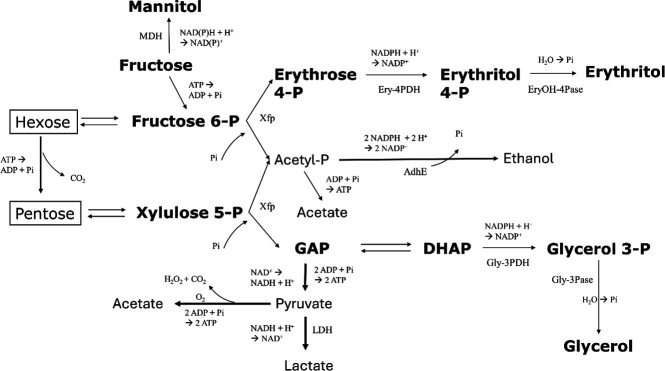
Proposed pathways for the biosynthesis of erythritol, glycerol, and mannitol by heterofermentative lactic acid bacteria, starting from hexose and pentose monosaccharides (based on references 12, 17, 28, 29). Adh, bifunctional acetaldehyde/ethanol dehydrogenase; Ery-4PDH, erythrose 4-phosphate dehydrogenase; EryOH-4Pase, erythritol 4-phosphate phosphatase; Glyc-3PDH, glycerol 3-phosphate dehydrogenase; Glyc-3Pase, glycerol 3-phosphate phosphatase; LDH, lactate dehydrogenase; MDH, mannitol dehydrogenase; Xfp, phosphoketolase.

It has been shown that in LAB species, such as *Leuconostoc mesenteroides* and *O. oeni*, ethanol biosynthesis represents a limiting step during hexose fermentation, as it is acetyl-CoA- and, thus, HSCoA-dependent (in turn pantothenate-dependent), and, therefore, excess NAD(P)H is reoxidized by the HSCoA-independent production of erythritol when pantothenate is lacking (12). The validity of this mechanism has been supported by nuclear magnetic resonance (NMR) spectra of a ^13^C-marked glucose metabolism (25). To this end, fructose 6-phosphate is first converted into erythrose 4-phosphate by a D-xylulose 5-phosphate/D-fructose 6-phosphoketolase (Xfp of *O. oeni* and *Bifidobacterium*, EC 4.1.2.22), then reduced into erythritol 4-phosphate, and finally converted into erythritol after removal of the phosphate group (Fig. 1). The enzymes responsible for the second and third steps of this biosynthesis pathway have not been identified in LAB yet, and thus the protein sequences are not available for genome mining (3, 12). These activities are probably executed by the enzymes erythritol 4-phosphate dehydrogenase for the second step and erythritol 4-phosphate phosphatase for the third step of this pathway. *Oenococcus oeni* produces erythritol from glucose under anaerobic conditions, but not from fructose or ribose. In the absence of oxygen, the NADP^+^-dependent glucose 6-phosphate dehydrogenase enzyme cannot process all available glucose 6-phosphate molecules, leaving more glucose 6-phosphate for conversion into fructose 6-phosphate. Concerning fungi, erythrose reductase has been suggested as the key enzyme for the production of erythritol by *Trichosporonoides megachiliensis*, which converts D-erythrose into *meso*-erythritol using NADPH as a reductant (26). Furthermore, the yeast *Yarrowia lipolytica* produces erythritol and mannitol simultaneously, whereby their ratios depend on the presence of glycerol and salt (27). The addition of salt improves erythritol biosynthesis, and simultaneously inhibits mannitol biosynthesis, indicating gene regulation by external factors.

The aim of the present study was to characterize the production of erythritol by the sourdough strains *L. fermentum* IMDO 130101 and IMDO TC9L10, in the context of their broader fermentation potential, given the assumed erythritol biosynthesis by *L. fermentum* during sourdough productions. Therefore, starter culture-initiated sourdough productions were performed, followed by fermentation processes in a liquid wheat sourdough simulation medium (WSSM). Also, the enzymatic pathways for erythritol production were probed *in silico*.

## RESULTS

### *Limosilactobacillus fermentum***-**initiated wheat sourdough productions

**Fig 2:**
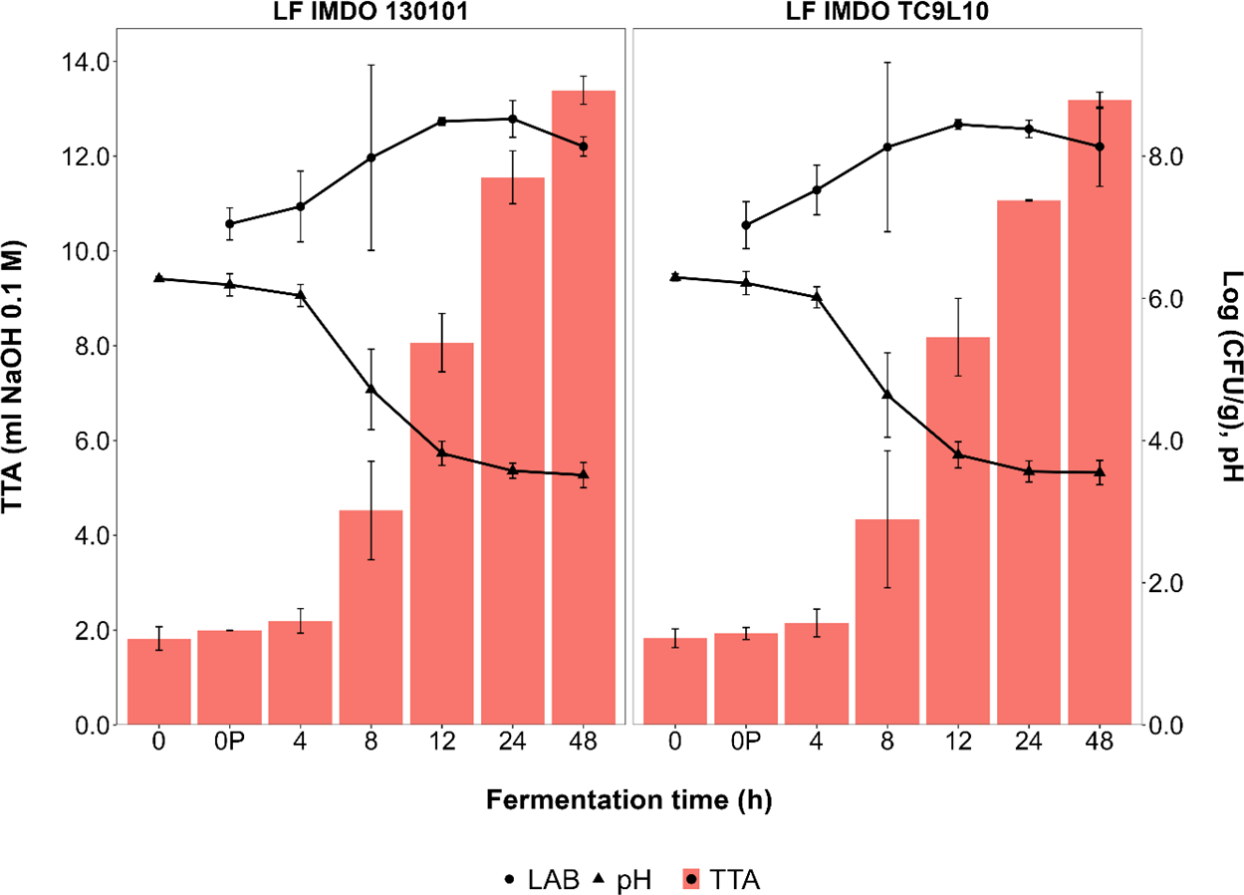
Course of the pH (triangles, right axis), total titratable acidity (TTA; bars, left axis), and counts [log (CFU/g)] of the presumptive lactic acid bacteria (circles, right axis) over 48 h of wheat sourdough productions carried out with *Limosilactobacillus fermentum* IMDO 130101 and IMDO TC9L10.

The dynamics of the wheat sourdough productions carried out with two strains of *L. fermentum* (IMDO 130101 and IMDO TC9L10), both originally isolated from sourdoughs, was assessed (Fig. 2). The pH and TTA values progressed in a similar way, reaching final values of 3.52 and 3.55 for the pH courses, and 13.4 and 13.2 mL for the TTA courses, respectively, after 48 h of fermentation. The maximum cell densities reached 8.5 log (CFU/g) for both *L. fermentum* strains after 12 h of fermentation. Yeasts were below the detection limit on YPG agar medium.

**Fig 3:**
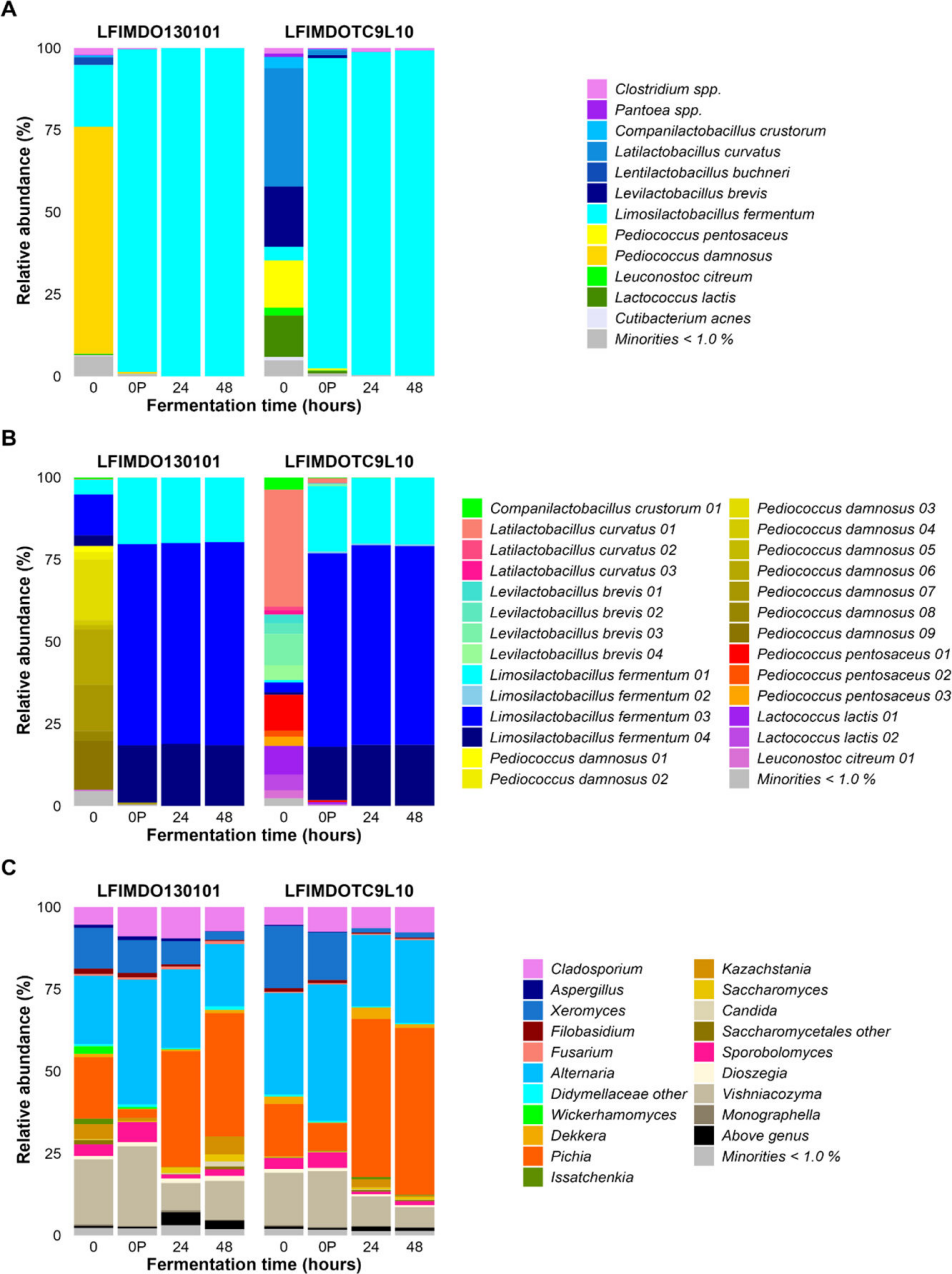
Culture-independent microbial community dynamics expressed as relative abundances (% of the total number of sequence reads), based on the amplicon sequence variants (ASVs) of the full-length 16S rRNA gene for bacteria (**A**), those constrained to the Lactobacillales order (**B**), and the internal transcribed spacer (ITS) region (ITS1) of the fungal rRNA transcribed unit (**C**), during the course of wheat sourdough productions carried out with *Limosilactobacillus fermentum* IMDO 130101 and IMDO TC9L10.

The culture-independent assessment of the microbial community dynamics revealed that, immediately after inoculation (0’ h), *L. fermentum* was the prevalent species present in all wheat sourdough productions carried out until 48 h of fermentation (Fig. 3). Furthermore, amplicon sequence variants (ASVs) belonging to *L. fermentum* present in the wheat sourdough samples matched the corresponding sequences in the genomes of the strains used, indicating successful inoculation and competitiveness of these strains. Regarding the fungal community dynamics, the main course was that of a slight increase of the genus *Pichia*, as well as a decrease of the genera *Alternaria*, *Xeromyces*, and *Vishniacozyma*, which was not reflected in the viable counts on YPG agar medium, indicating left-overs of DNA of these fungi in the samples examined.

**Fig 4:**
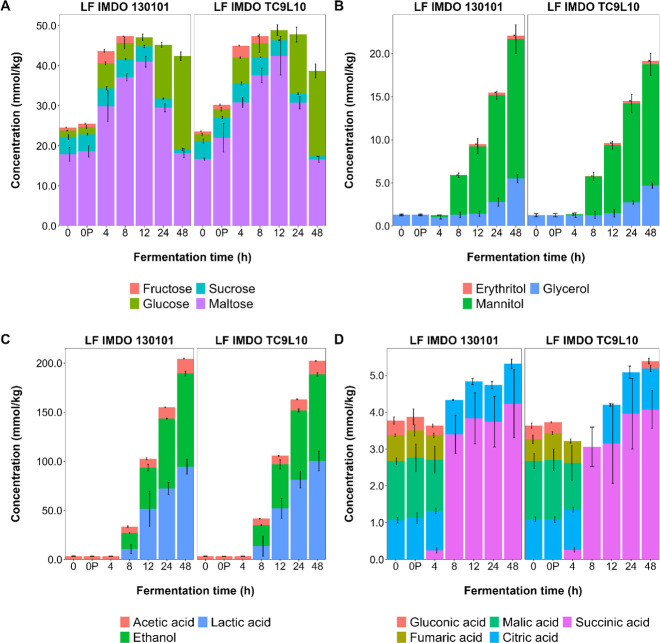
Dynamics of the concentrations (mmol/kg) of the mono- and disaccharides (**A**), sugar alcohols (**B**), lactic acid, ethanol, and acetic acid (**C**), and other organic acids (**D**) during the course of wheat sourdough productions carried out with *Limosilactobacillus fermentum* IMDO 130101 and IMDO TC9L10.

During the wheat sourdough productions carried out, the carbohydrates were metabolized to organic acids, ethanol, and sugar alcohols (Fig. 4), which became noticeable after 4–8 h of fermentation. Upon fermentation, the fructose concentrations dropped below the limit of quantification after 8 h, whereas the sucrose concentrations decreased to 0.8 mmol/kg and 1.0 mmol/kg for *L. fermentum* IMDO 130101 and IMDO TC9L10, respectively. The glucose concentrations increased 10-fold, indicating a continuous release from maltose that was not taken up directly. The concentrations of malic acid and fumaric acid were rapidly depleted, within 4–8 h of fermentation. After 48 h of fermentation, equimolar quantities of lactic acid and ethanol were produced by both strains. Regarding the sugar alcohol dynamics, the same trends were found for *L. fermentum* IMDO 130101 and IMDO TC9L10, with small yet non-significant differences with respect to the absolute quantities produced for mannitol (16.2 and 14.1 mmol/kg, respectively), glycerol (5.5 and 4.7 mmol/kg, respectively), and erythritol (0.41 and 0.39 mmol/kg, respectively), indicating a flux through different branches of carbohydrate breakdown for redox balancing simultaneously. Sorbitol was not found. Citric acid was present in the unfermented doughs, but its concentration was not altered during the course of the wheat sourdough productions. Furthermore, up to 4.0 mmol/kg of succinic acid was produced by both strains.

### *In silico* analysis of *Limosilactobacillus fermentum* genomes

#### Erythritol biosynthesis

The search for protein sequences related to the erythritol biosynthesis pathway within the order of the Lactobacillales in public databases did not yield results. When the search parameters were broadened to all bacteria present in these databases, a small number of candidate protein sequences were found for *Brucella abortus* (UniProt accession numbers Q2YIQ6 and Q2YIQ1), *Brucella melitensis* (AAL53671.1), *Mycolicibacterium smegmatis* (A0QXD8), and *Ensifer fredii* (AAQ87123.1). Among those, only the accession numbers reported in Tables 1 and 2 resulted in positive hits for *L. fermentum*. However, none of the *L. fermentum* IMDO 130101- and TC9L10-coding sequences that yielded positive hits included the terms “erythritol” or “erythrose” in their annotations. The most relevant alignments were related to the metabolism of other sugar alcohols, such as glycerol and mannitol, and the use of zinc or NADP^+^ as cofactors.

**TABLE 1:** Most relevant hits found in the genome of *Limosilactobacillus fermentum* IMDO 130101 based on protein sequences related to the biosynthesis of erythritol used as BLAST queries

Accession number	Microorganism	Annotated function query	Protein size (AA)	Annotated function *L. fermentum* IMDO 130101	Identity/ similarity (%)	Query coverage (%)	Protein locus tag
Q2YIQ6	*Brucella abortus*	L-Erythrulose-1-phosphate isomerase	256	Triosephosphate isomerase	33/58	96.09	SNX31105.1
Q2YIQ6	*B. abortus*	L-Erythrulose-1-phosphate isomerase	256	Triosephosphate isomerase	33/54	96.09	SNX31055.1
Q2YIQ1	*B. abortus*	Erythritol kinase	520	Xylulose kinase	26/44	98.08	SNX31659.1
Q2YIQ1	*B. abortus*	Erythritol kinase	520	Xylulose kinase	24/33	95.38	SNX31092.1
Q2YIQ1	*B. abortus*	Erythritol kinase	520	Xylulose kinase	23/42	97.88	SNX32502.1
Q2YIQ1	*B. abortus*	Erythritol kinase	520	Glycerol kinase	21/37	88.85	SNX31728.1
A0QXD8	*Mycolicibacterium smegmatis*	Erythritol/L-threitol dehydrogenase	362	Zinc-dependent alcohol dehydrogenase family protein	25/44	96.7	SNX32568.1
A0QXD8	*M. smegmatis*	Erythritol/L-threitol dehydrogenase	362	Mannitol 2-dehydrogenase	25/44	92.27	SNX31565.1
A0QXD8	*M. smegmatis*	Erythritol/L-threitol dehydrogenase	362	2,3-Butanediol dehydrogenase	25/43	92.27	SNX32256.1
A0QXD8	*M. smegmatis*	Erythritol/L-threitol dehydrogenase	362	Alcohol dehydrogenase (AdhP)	32/48	96.41	SNX31414.1
A0QXD8	*M. smegmatis*	Erythritol/L-threitol dehydrogenase	362	NAD(P)-dependent alcohol dehydrogenase	25/41	75.14	SNX30849.1

**TABLE 2:** Most relevant hits found in the genome of *limosilactobacillus fermentum* IMDO TC9L10 based on protein sequences related to the biosynthesis of erythritol used as BLAST queries

Accession number	Microorganism	Annotated function query	Protein size (AA)	Annotated function *L. fermentum* IMDO TC9L10	Identity/ similarity (%)	Query coverage (%)	Protein locus tag
Q2YIQ6	*Brucella abortus*	L-Erythrulose-1-phosphate isomerase	256	Triosephosphate isomerase	33/58	96.09	ODKFFKCD_00481
Q2YIQ6	*B. abortus*	L-Erythrulose-1-phosphate isomerase	256	Triosephosphate isomerase	33/54	96.09	ODKFFKCD_00426
Q2YIQ1	*B. abortus*	Erythritol kinase	520	Xylulose kinase	26/44	98.08	ODKFFKCD_01107
Q2YIQ1	*B. abortus*	Erythritol kinase	520	Xylulose kinase	24/33	95.38	ODKFFKCD_00466
Q2YIQ1	*B. abortus*	Erythritol kinase	520	Xylulose kinase	23/42	97.88	ODKFFKCD_02034
Q2YIQ1	*B. abortus*	Erythritol kinase	520	Glycerol kinase	21/37	88.85	ODKFFKCD_01182
A0QXD8	*Mycolicibacterium smegmatis*	Erythritol/L-threitol dehydrogenase	362	Zinc-dependent alcohol dehydrogenase family protein	25/44	96.7	ODKFFKCD_02100
A0QXD8	*M. smegmatis*	Erythritol/L-threitol dehydrogenase	362	Mannitol 2-dehydrogenase	25/44	92.27	ODKFFKCD_01009
A0QXD8	*M. smegmatis*	Erythritol/L-threitol dehydrogenase	362	2,3-Butanediol dehydrogenase	25/43	92.27	ODKFFKCD_01757
A0QXD8	*M. smegmatis*	Erythritol/L-threitol dehydrogenase	362	Alcohol dehydrogenase (AdhP)	32/48	96.41	ODKFFKCD_00845
A0QXD8	*M. smegmatis*	Erythritol/L-threitol dehydrogenase	362	NAD(P)-dependent alcohol dehydrogenase	25/41	75.14	ODKFFKCD_02100

#### Genomic comparison

Since both *L. fermentum* strains tested produced a similar metabolic output during the starter culture-initiated wheat sourdough productions, a genomic comparison of these two strains was carried out with DNAdiff (MUMer3). The *L. fermentum* IMDO 130101 genome comprised 2,089,202 bases, whereas that of *L. fermentum* IMDO TC9L10 consisted of 2,089,036 bases. Their average nucleotide identity (ANI) values were 99.99%. *Limosilactobacillus fermentum* IMDO 130101 possessed two more GAGCA repeats in a series of such repeats in an intergenic region near position 735,520, and a 159-nucleotide duplicated sequence that was only found in one copy within the genome of *L. fermentum* IMDO TC9L10 at positions 1,075,514–1,075,714. Based on the high degree of similarity between the *L. fermentum* IMDO 130101 and IMDO TC9L10 genomes, further experiments were only conducted with the strain *L. fermentum* IMDO 130101, given the acquired knowledge for this strain in the past.

### Fermentation processes in wheat sourdough simulation medium with *Limosilactobacillus fermentum* IMDO 130101

To further investigate the erythritol biosynthesis potential of *L. fermentum* IMDO 130101, fermentation processes were carried out in WSSM, both at small- and medium-scale.

#### Small-scale fermentation processes

**Fig 5:**
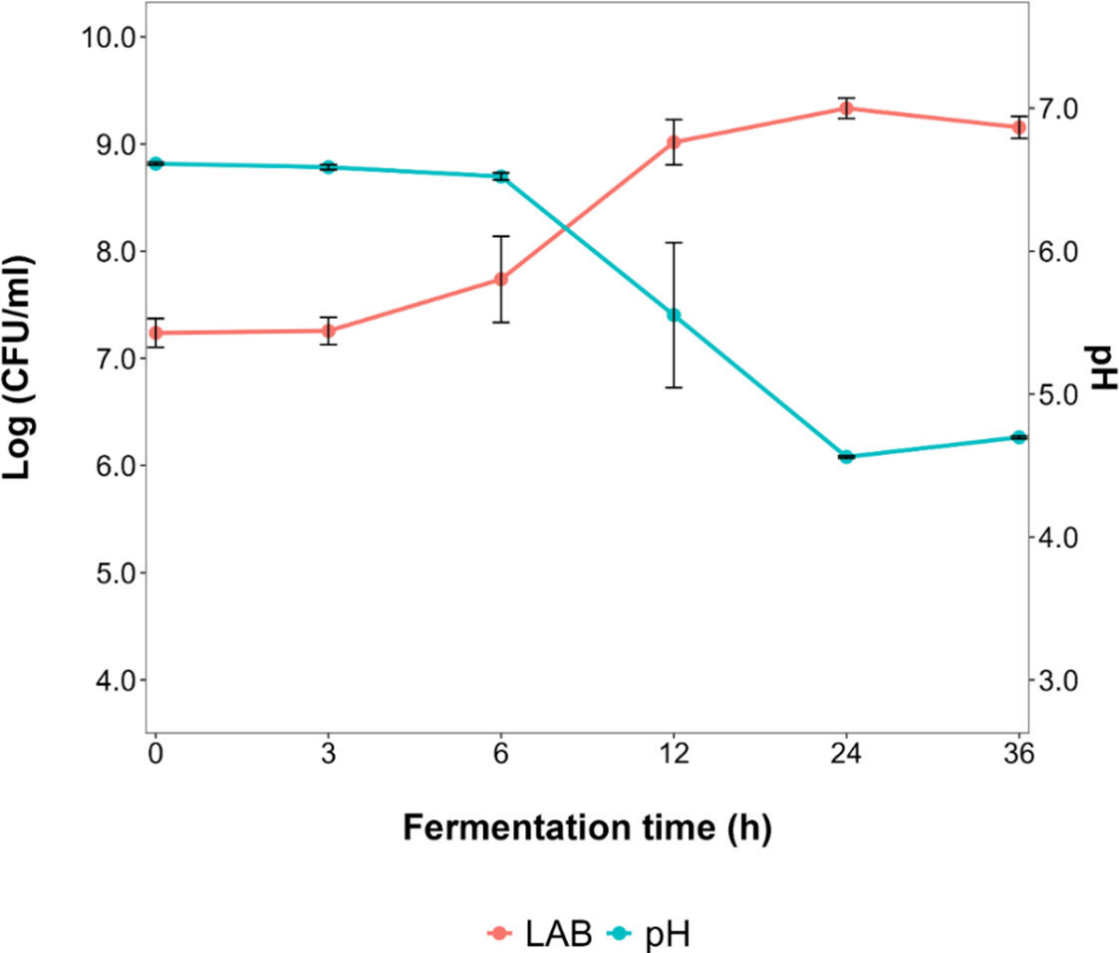
Course of the pH and bacterial counts (log [CFU/mL]) during fermentation processes with *Limosilactobacillus fermentum* IMDO 130101 in 100 mL of wheat sourdough simulation medium (WSSM).

For the small-scale fermentation processes, the fermentation dynamics of *L. fermentum* IMDO 130101 in WSSM were followed at 100 mL scale in glass bottles (Fig. 5). After 6 h of fermentation, the pH decreased and eventually reached a value of 4.5 after 24 h, the time point at which a maximum cell dry mass was obtained and when the viable counts amounted to 9.3 log (CFU/mL).

**Fig 6:**
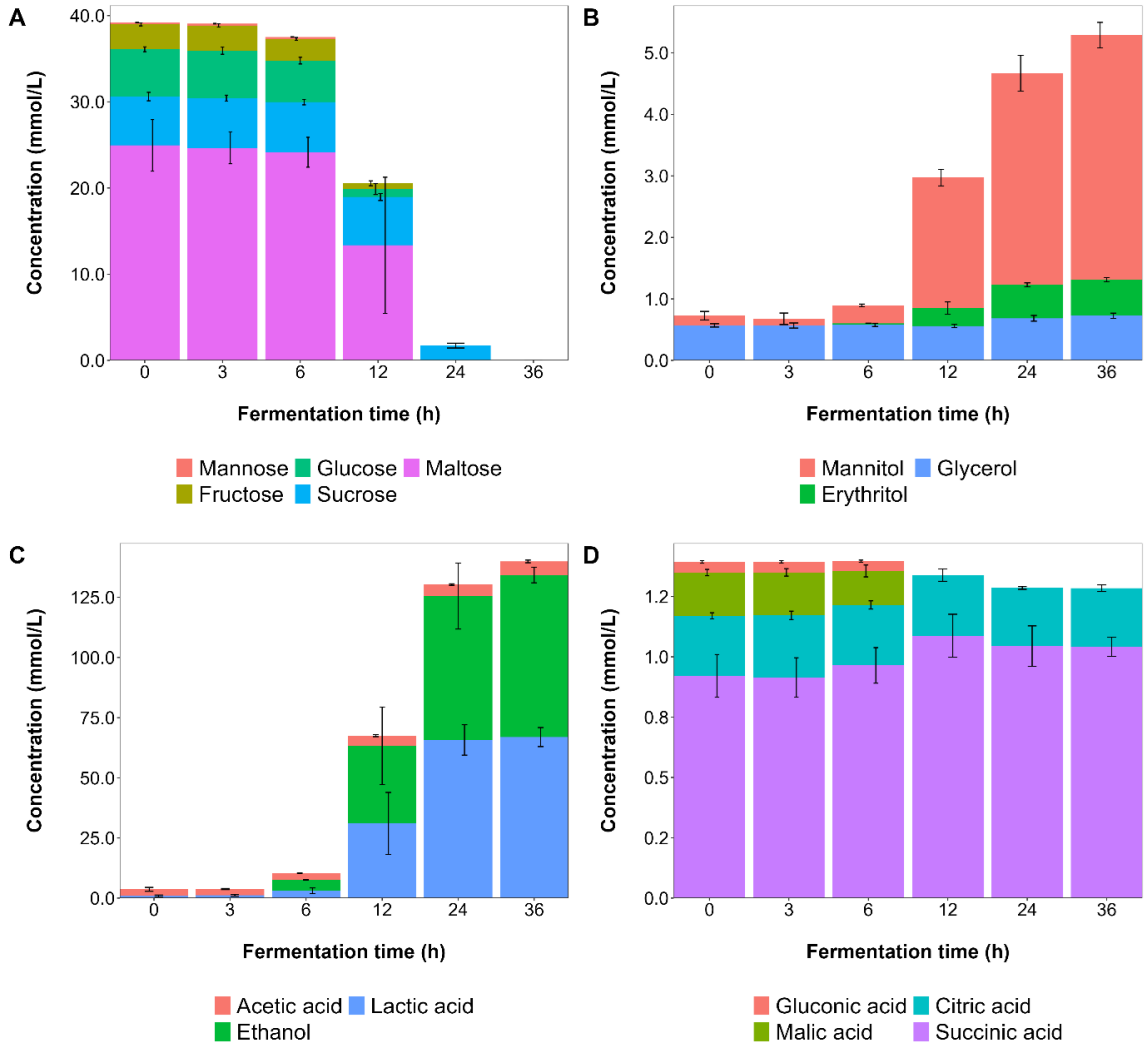
Course of the concentrations of the mono- and disaccharides (**A**), sugar alcohols (**B**), lactic acid, ethanol, and acetic acid (**C**), and other organic acids (**D**) during fermentation processes with *Limosilactobacillus fermentum* IMDO 130101 in 100 mL of wheat sourdough simulation medium (WSSM).

The carbohydrates were depleted in the order of glucose, fructose, maltose, and sucrose. They were metabolized to ethanol (67.4 mM), lactic acid (66.9 mM), acetic acid (5.7 mM), mannitol (3.81 mM), erythritol (0.59 mM), glycerol (0.16 mM), and succinic acid (0.12 mM). Sorbitol was not found. Citric acid was present in the medium since the start but was not metabolized, whereas the initial malic acid concentration was depleted after 12 h of fermentation (Fig. 6).

The total amount of carbon sources present in the WSSM corresponded with 420 mM of carbon equivalents, whereas the total of metabolites produced amounted to 365 mM of carbon equivalents (not taking into account carbon dioxide production, as it was not measured during the small-scale WSSM fermentation processes), representing a carbon recovery of 87%.

#### Medium-scale fermentation processes

**Fig 7:**
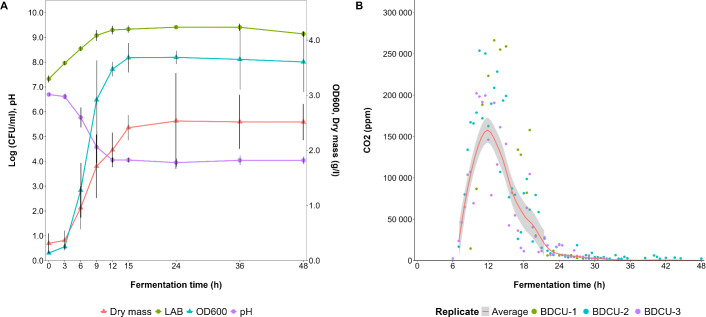
Fermentation dynamics of *Limosilactobacillus fermentum* IMDO 130101 in 2 L of wheat sourdough simulation medium (WSSM), represented as biomass (cell dry mass, g/L), counts (log [CFU/mL]), optical density (OD_600_), and pH (**A**). The production of carbon dioxide was followed online (**B**).

For the medium-scale fermentation processes, the fermentation dynamics of *L. fermentum* IMDO 130101 in WSSM was followed at 2-L scale in fermentation vessels (Fig. 7). After 3-6 h of fermentation, the pH decreased to ultimately reach a value of 4.0 after 12 h. Similarly, the optical density at 600 nm (OD_600_) and bacterial counts reached maximum values after 12 h of fermentation. At this time point, also the carbon dioxide production was maximal, decreasing afterward, and becoming negligible after 24 h of fermentation. The total cell dry mass reached its maximum value (2.5 g/L) after 24 h of fermentation.

**Fig 8:**
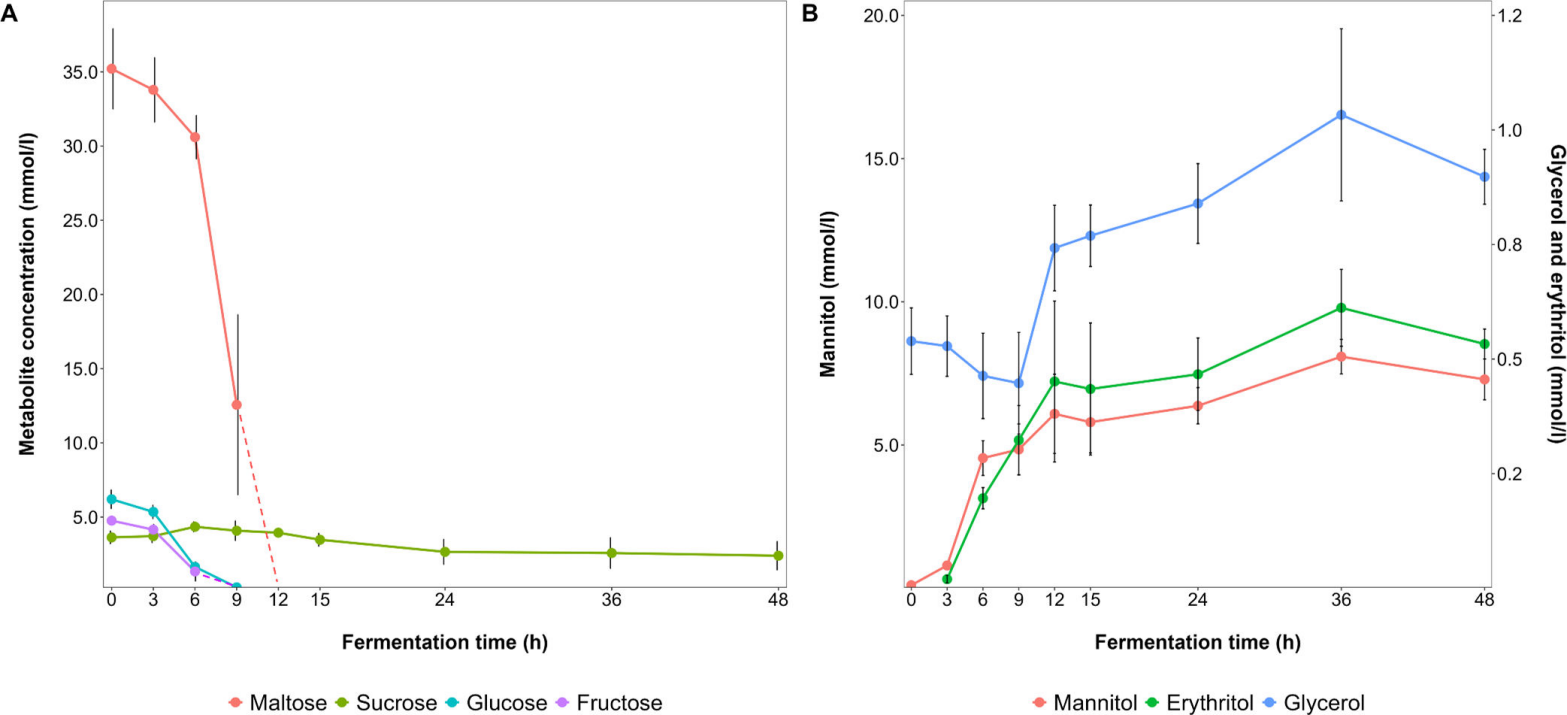
Dynamics of the concentrations (mmol/L) of mono- and disaccharides (**A**) and sugar alcohols (**B**) during fermentation processes with *Limosilactobacillus fermentum* IMDO 130101 in 2 L of wheat sourdough simulation medium (WSSM).

**Fig 9:**
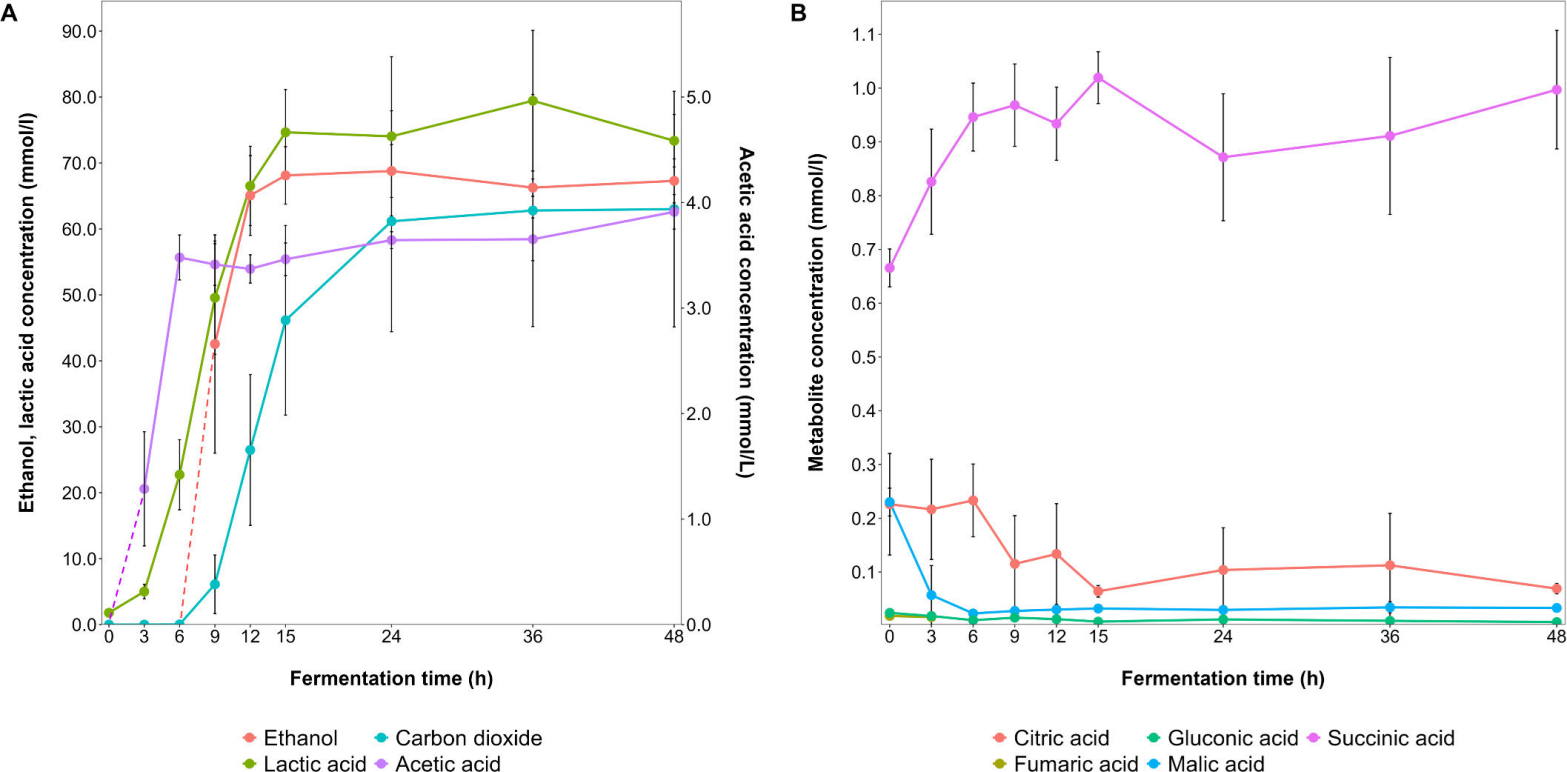
Dynamics of the concentrations (mmol/L) of ethanol, lactic acid, and acetic acid (**A**) and other organic acids (**B**) during fermentation processes with *Limosilactobacillus fermentum* IMDO 130101 in 2 L of wheat sourdough simulation medium (WSSM).

Carbohydrate consumption occurred in such an order that glucose and fructose were depleted after 9 h and maltose after 12 h of fermentation (Fig. 8A). Sucrose was not completely depleted after 48 h of fermentation, as only about 50% of its initial concentration was consumed. During fermentation, lactic acid (73.4 mM, Fig. 9A), ethanol (67.3 mM, Fig. 9A), carbon dioxide (63.0 mM, Fig. 7B), mannitol (7.2 mM, Fig. 8B), acetic acid (3.9 mM, Fig. 8A), erythritol (0.53 mM, Fig. 8B), and glycerol (0.36 mM, Fig. 8B) were produced. Sorbitol was not found. Most of these metabolite productions took place between 3 and 12 h of fermentation, after which time point sucrose remained available. Hence, the concentrations of lactic acid, mannitol, and erythritol still increased until the end of the fermentation processes, but at a reduced rate. All acetic acid production took place within the first 6 h of fermentation, halting earlier than the production of the other metabolites.

In total, 503.8 mM carbon equivalents were present at the beginning of the WSSM fermentation processes, of which 28.7 mM was still present at the end in the form of sucrose. The carbon equivalents of the metabolites produced were 466.9 mM, which took carbon dioxide production into account and yielded a final carbon recovery of 98%.

### Comparison of metabolite production for all fermentation processes carried out

The production of sugar alcohols by the *L. fermentum* strains tested during the wheat sourdough productions carried out differed only slightly (Table 3). In contrast, the concentrations of the sugar alcohols produced differed when *L. fermentum* IMDO 130101 was grown at small- or medium-scale during the WSSM fermentation processes. For both glycerol and mannitol, the concentrations varied, while the concentrations of erythritol were relatively the same (0.4–0.6 mM). Regarding the ratios of the sugar alcohols produced, no major trends occurred. Yet, the ratio of erythritol to mannitol was the most consistent, albeit with a different value for the different fermentation conditions, indicating a possible relationship between the production of these two sugar alcohols. When taking the molar conversions of glucose equivalents into metabolites into account, after deducting those used for mannitol production, near equimolar concentrations of lactic acid, ethanol, and carbon dioxide were produced from glucose (Table 4). More acetic acid and glycerol were produced during the wheat sourdough productions compared to the fermentation processes in WSSM. There seemed to be a trend toward slightly more erythritol production when less acetic acid and glycerol were produced. Also, erythritol, glycerol, and mannitol production continued during the stationary growth phase (Fig. 8 and 9).

**TABLE 3:** Production of sugar alcohols and their relative ratios by *Limosilactobacillus fermentum* IMDO 130101 and IMDO TC9L10 during wheat sourdough production (both strains) and in wheat sourdough simulation medium (WSSM; only *L. fermentum* IMDO 130101)^*a*^

Strain	Time (h)	Glycerol (mM)	Mannitol (mM)	Erythritol (mM)	Erythritol/mannitol	Erythritol/glycerol	Glycerol/mannitol
Wheat sourdough productions							
IMDO 130101	4	0.00	0.21	0.00	NA	NA	NA
	8	0.01	4.60	0.03	0.01	4.88	0.00
	12	0.12	7.84	0.25	0.03	2.03	0.02
	24	1.49	12.42	0.30	0.02	0.20	0.12
	48	4.21	16.20	0.41	0.03	0.01	0.26
IMDO TC9L10	4	0.01	0.11	0.00	NA	NA	0.06
	8	0.03	4.48	0.06	0.01	1.80	0.01
	12	0.23	7.89	0.26	0.03	1.14	0.03
	24	1.49	11.47	0.31	0.03	0.21	0.13
	48	3.43	14.08	0.39	0.03	0.11	0.24
Small-scale WSSM fermentation processes							
IMDO 130101	3	0.00	0.00	0.00	NA	NA	NA
	6	0.01	0.00	0.03	NA	2.77	NA
	9	0.01	0.13	0.03	0.20	2.54	0.07
	12	0.01	1.96	0.29	0.15	29.10	0.01
	24	0.11	3.28	0.54	0.17	4.77	0.04
	36	0.16	3.81	0.59	0.15	3.78	0.04
Medium-scale WSSM fermentation processes							
IMDO 130101	3	0.00	0.69	0.02	0.03	NA	NA
	6	0.00	4.44	0.20	0.04	NA	NA
	9	0.00	4.74	0.32	0.07	NA	NA
	12	0.20	5.98	0.45	0.08	2.22	0.03
	15	0.23	5.69	0.44	0.08	1.89	0.04
	24	0.30	6.27	0.47	0.08	1.55	0.05
	36	0.49	7.98	0.61	0.08	1.24	0.06
	48	0.36	7.19	0.53	0.07	1.49	0.05

^*a*^ NA, not applicable.

**TABLE 4:** Approximate conversions of glucose equivalents and fructose equivalents by *Limosilactobacillus fermentum* IMDO 130101 and IMDO TC9L10 during wheat sourdough production (both strains) and in wheat sourdough simulation medium (WSSM; only *L. fermentum* IMDO 130101)^*a*^

*Limosilactobacillus* fermentum strain	Glucose equivalents (mM)	Fructose equivalents (mM)		Lactic acid (mM)	Ethanol (mM)	Carbon dioxide (mM)	Acetic acid (mM)	Mannitol (mM)	Glycerol (mM)	Erythritol (mM)
Sourdough productions (48 h)										
IMDO 130101	NA	5.2	→	95.0	95.0	ND	12.0	16.2	4.2	0.4
IMDO TC9L10	NA	5.8	→	100.5	88.0	ND	10.5	14.1	3.5	0.4
Small-scale WSSM fermentation process (36 h)										
IMDO 130101	61.5	8.6	→	67.0	67.5	ND	3.0	3.8	0.2	0.6
Medium-scale WSSM fermentation process (48 h)										
IMDO 130101	77.0	6.0	→	73.5	67.0	63.0	4.0	7.2	0.4	0.5

^*a*^ NA, not available; ND, not determined.

## DISCUSSION

The present study confirmed the presumed potential of erythritol biosynthesis by certain LAB species, in particular *L. fermentum* during wheat sourdough production, and showed the erythritol biosynthesis dynamics of *L. fermentum* IMDO 130101 in a WSSM. The biosynthesis of erythritol by LAB has been ascribed to the metabolism of hexoses (and pentoses, such as arabinose and xylose, to recuperate NAD^+^/NADP^+^ when not enough ethanol can be produced and, hence, excess NAD(P)H + H^+^ has to be oxidized for redox balancing (28). Consequently, under ethanol production limitation conditions, erythritol biosynthesis can function as an alternative NAD(P)H + H^+^ regenerative pathway in heterofermentative LAB species, such as *L. fermentum*. However, during the fermentation processes of the present study, ethanol production limitation did not occur, indicating additional or other factors that might influence branching of the glucose breakdown pathway. Indeed, erythritol, mannitol, and glycerol production continued during the stationary growth phase, when the osmotic stress was lower, indicating their main involvement in redox balancing. Further, an alignment search through BLASTp during the present study could not identify genes encoding erythritol-producing enzymes in either *L. fermentum* IMDO 130101 or IMDO TC9L10. Indeed, the enzymes responsible for the conversion of erythrose 4-phosphate into erythritol 4-phospate, and its subsequent conversion into phosphate-free erythritol, have not yet been characterized (12). Therefore, the enzymes involved in this process are likely distinct from the ones that were used for the BLASTp queries of the present study.

One possibility to explain the erythritol biosynthesis potential of *L. fermentum* is that the responsible dehydrogenase and phosphatase enzymes are monofunctional and have not received attention to be identified as erythritol biosynthesis-related enzymes through fundamental biochemical studies. Another possibility is that the biosynthesis of erythritol is carried out in LAB by bifunctional enzymes, whose main function is the production of mannitol or glycerol. However, studies on the biochemical characteristics of the mannitol biosynthesis-related enzymes have not considered the possibility of their functioning as erythritol-producing enzymes. Yet, *L. fermentum* IMDO 130101 contains a gene (LF130101_2113; 30) that encodes a protein with 88% identity and 94% similarity to a mannitol 2-dehydrogenase from *Limosilactobacillus reuter*i (LRMDH; 31). The latter enzyme reduces D-fructose to D-mannitol and has a preference for NADPH + H^+^ over NADH + H^+^. This preference is even greater when performing oxidation of D-mannitol to D-fructose. The enzyme is not active on D-fructose 6-phosphate, D-mannose, L-arabinose, D-sorbitol, D-arabitol, L-arabitol, xylitol, adonitol, L-sorbose, ribitol, or 2-propanol, and has a very low activity on D-fructose 1-phosphate. Erythritol, however, was not tested. Also, Zn^2+^ is a cofactor of this enzyme. A homolog of the *M. smegmatis* erythritol/L-threitol dehydrogenase (SwissProt accession number A0QXD8) has been annotated in *L. fermentum* IMDO 130101 (30) as a zinc-dependent alcohol dehydrogenase family protein, suggesting a similar function.

Proteins, such as LF130101_2113 (WP_104878727.1, 336 AA; 29), are “threonine dehydrogenase-like” enzymes. Indeed, *O. oeni* is a LAB species known to include mannitol- and erythritol-producing strains, and the type strain of this species encodes a protein (WP_002819553.1) with 64% identity and 80% similarity to LF130101_2113 that belongs to this “threonine dehydrogenase-like” group. The involvement of these and other enzymes/proteins in erythritol biosynthesis should be further investigated.

Within the *L. fermentum* IMDO 130101 genome, another potentially interesting protein has been uncovered, namely, a “mannitol dehydrogenase family” protein (WP_104878684.1, 548 AA; 29) that is similar to a mannitol 2-dehydrogenase from *Aspergillus fischeri*. According to the conserved domains database (CDD), this protein is akin to mannitol-1-phosphate/altronate reductase (MtlD) and contains a long-chain mannitol dehydrogenase C-terminal domain, as well as a very weakly supported mannitol dehydrogenase Rossmann domain at the N-terminus. This type of enzyme is independent of Zn^2+^. However, it potentially plays a role in fructuronate metabolism. Very few *O. oeni* genomes contain a gene encoding a homologous protein (only two RefSeq protein hits, originating from five genomes, with at most 56% identity and 72% similarity), and neither does *F. florum*. As this enzyme is not present in many *O. oeni* or any *F. florum* strains, it is unlikely to be involved in erythritol biosynthesis.

In *Brucella*, erythritol can serve as a carbon source (32). It is first phosphorylated to L-erythritol 4-phosphate, followed by oxidation to L-3-tetrulose 4-phosphate and three isomerization steps to form D-3-tetrulose 4-phosphate, D-erythrulose 4-phosphate, and D-erythrose 4-phosphate consecutively. In principle, LAB could possess these isomerases to catalyze the conversion of erythrose 4-phosphate into erythrulose 4-phosphate, D-3-tetrulose 4-phosphate, and L-3-tetrulose 4-phosphate, followed by a reduction to L-erythritol 4-phosphate and subsequent dephosphorylation, yielding free erythritol. This is, however, unlikely given the lack of highly identical BLASTp hits or hits with sufficient identity and only generally defined functions for the *L. fermentum* IMDO 130101-encoded proteins found.

Finally, in the yeast species *Y. lipolytica*, the route via the PPP, whereby erythrose is converted into erythritol by means of erythrose reductase activity, is followed. However, this route is excluded for LAB, as there is no PPP present, nor is the gene encoding this enzyme present, based on the gene annotation of the *L. fermentum* IMDO 130101 genome. Similarly, a BLASTn search of the erythrose reductase form *Y. lipolytica* (nucleotide NCBI database accession number JX885666.1) against the genome of *L. fermentum* IMDO 130101 did not result in any alignment. However, a BLASTn alignment of the erythrose reductase from a *Bacillus* sp. (nucleotide NCBI database accession number PP967939.1) against the genome of *L. fermentum* IMDO 130101 gave 100% coverage and 81% identity, the latter low value likely pointing toward another type of reductase.

Alternatively, the fact that the production of erythritol was nearly constant across the different fermentation processes carried out (slightly less during the wheat sourdough productions) might point to an unknown factor limiting the activation of the erythritol biosynthesis pathway. Further, it was likely that most of the glucose was metabolized to ethanol and lactate, as near equimolar concentrations of lactic acid, ethanol, and carbon dioxide were produced (carbon dioxide production was only verified for the medium-scale WSSM fermentation processes). However, when less acetic acid and glycerol were produced, the branch toward erythritol biosynthesis was more active. This might indicate a regulation of the bifunctional phosphoketolase catalyzing the conversion of either fructose 6-phosphate into erythrose 4-phosphate and acetyl-phosphate, or xylulose 5-phosphate into glyceraldehyde 3-phosphate and acetyl-phosphate.

It is further known that, when the pyruvate, citrate, oxygen, or fructose availability as internal or external electron acceptors is limited, alternative low-activity pathways become active to sustain additional LAB growth. Since the low concentration of citrate, which should provide the cell with extra pyruvate intracellularly, was not metabolized, while fructose was reduced to mannitol, further growth support could be provided by oxygen. Although the oxygen concentration was not determined, it was likely that it was partially depleted during the fermentation processes in WSSM, as no aeration was provided, whereas during the wheat sourdough productions some oxygen was included through mixing upon sampling, perhaps reflecting the higher and continuous acetic acid production during the latter processes. The lesser production of glycerol and acetate relative to that of erythritol during the WSSM fermentation processes may be explained by the oxygen tension too, as is the case for several strains of *O. oeni* (17). Furthermore, in *O. oeni*, erythritol and acetate production from glucose is enhanced in the absence of pantothenate, which is a co-factor required in the biosynthesis of CoA. Due to the limited availability of CoA, phosphotransacetylase and acetaldehyde dehydrogenase from the ethanol pathway are less active, causing a shift to erythritol, acetate, and glycerol biosynthesis (16). During the *L. fermentum* IMDO 130101 fermentation processes in WSSM, the production of acetic acid was halted earlier than that of erythritol. The production of erythritol followed a similar course as that of glycerol and mannitol, reaching a maximum production when all monosaccharides were depleted. This indicated that when erythritol biosynthesis was favored, the production of glycerol, and to a lesser extent that of acetate, was disfavored.

Finally, the fact that the erythritol-to-mannitol ratio remained constant within a fermentation process, and the fact that both sugar alcohols were produced when the glucose concentrations diminished and the consumption of fructose started were further in line with the idea that erythritol is produced because of the secondary activity of existing enzymes, namely xylulose 5-phosphate phosphoketolase and mannitol 2-dehydrogenase (17). Imported fructose could then be processed in the heterofermentative pathway, reduced into mannitol through the main activity of mannitol 2-dehydrogenase, or split into acetyl phosphate and erythrose 4-phosphate through the secondary activity of xylulose 5-phosphate phosphoketolase. Erythrose 4-phosphate could, in turn, be reduced to erythritol 4-phosphate through the secondary activity of the mannitol 2-dehydrogenase and then dephosphorylated by a phosphatase. This hypothesis again supported the bifunctionality of the enzymes involved.

In conclusion, *L. fermentum* IMDO 130101 could produce small quantities of erythritol in a wheat sourdough matrix, whereby its primary role was a need for redox balancing. When grown in a WSSM medium, the biosynthesis of erythritol was favored to the detriment of glycerol and acetate, but its production dynamics followed a similar course as that of mannitol and glycerol during the fermentation processes. Future research should focus on the production of erythritol by *L. fermentum* and other heterofermentative LAB species under different fermentation conditions, specifically assessing different carbohydrates separately. Besides, the identification of the enzymes responsible for the production of erythritol has still not been achieved. To this end, comparative genomics of erythritol-producing strains *versus* non-producing strains could lead to the identification of the genes responsible for erythritol biosynthesis. Transcriptomics might be of help in narrowing down the search and further validating the functions of the genes found.

## MATERIALS AND METHODS

### Strains, media, and inoculum build-up

The sourdough strains *Limosilactobacillus fermentum* IMDO 130101 and IMDO TC9L10 were used throughout this study. These strains were isolated from a bakery wheat sourdough (21), and a backslopped sourdough production performed with triticale flour (22), respectively. Both strains were stored at −80°C in modified de Man-Rogosa-Sharpe-5 (mMRS-5) medium (33), supplemented with 25% (v/v) glycerol as a cryoprotectant (Sigma-Aldrich, Saint Louis, Missouri, USA). Before use, the strains were grown in either mMRS-5 medium for the wheat sourdough production experiments or a wheat sourdough simulation medium (WSSM; 34, 35) for the fermentation experiments. The composition of WSSM was as follows (per liter): maltose (Merck, Darmstadt, Germany), 10.0 g; sucrose (Merck), 2.0 g; glucose (Merck), 0.50 g; fructose (Merck), 0.50 g; yeast extract (Merck), 12.0 g; wheat peptone (Merck), 12.0 g; KH_2_PO_4_ (Merck), 4.0 g; MgSO_4_.7H_2_O (VWR International, Darmstadt, Germany), 0.20 g; MnSO_4_.H_2_O (VWR International), 0.050 g; Tween 80 (Sigma-Aldrich), 1.0 mL; and a vitamin solution, (0.20 mg), including cobalamin, folic acid, nicotinamide, pantothenic acid, pyridoxal phosphate, and thiamine (all from Sigma-Aldrich).

The inoculum build-up was performed as follows. The strains were first transferred from the storage vial at −80°C to mMRS-5 agar medium, and the plates were incubated at 30°C for 48 h. A single colony was transferred to 10 mL of either mMRS-5 medium or WSSM and incubated at 30°C for 24 h. Afterward, fresh liquid medium was inoculated at 0.1% (v/v) and incubated at 30°C for 16 h. Before the inoculation in the flour-water mixture (FWM) for the wheat sourdough production experiments or in WSSM for the fermentation experiments, the inoculum was centrifuged (4,694×*g*, 10 min, 4°C) and resuspended in a sterile saline solution (0.85%, m/v, NaCl; Merck) twice to remove microbial metabolites that could affect the normal progression of the experiment.

### *In silico* analyses

To assess the genetic information related to erythritol biosynthesis, a term search in the RefSeq subset of the National Center for Biotechnology Information (NCBI) protein database (36, 37; URL, https://www.ncbi.nlm.nih.gov/protein; last accessed 3 March 2024) was performed. Protein sequences annotated as active on erythrose or erythritol were screened first in bacteria, and specifically in members of the Lactobacillales order (Table 5). These sequences were aligned against the genomes of *L. fermentum* IMDO 130101 (NCBI Accession Number GCF_900205745.1) and *L. fermentum* IMDO TC9L10 using BLASTp (European Nucleotide Archive of the European Bioinformatics Institute [ENA/EBI] Accession Number ERS16236875) (38). Only two candidate sequences were found within the Lactobacillales order, namely, one for *Streptococcus pyogenes* (protein accession number TYK92996.1) and another for *Enterococcus faecium* (PWQ89175). Upon further analysis, these sequences have turned out to be probable contaminations due to genome sequencing, originating from *Mezorhizobium* sp. and *Pseudomonas putida*, respectively.

**TABLE 5:** Protein sequences related to erythritol biosynthesis in bacteria and retrieved from the NCBI protein database (37)^*a*^

Annotated function	Protein size (AA)	Microorganism	Accession
Erythrose-4-phosphate dehydrogenase (partial)	140	*Streptococcus pyogenes* (likely a contaminant from *Mesorhizobium* sp.)	TYK92996.1
Erythrose-4-phosphate dehydrogenase (partial)	97	*Enterococcus faecium* (likely a contaminant from *Pseudomonas putida*)	PWQ89175
D-Erythrulose-4-phosphate isomerase	151	*Brucella abortus*	Q9ZB26
L-Erythrulose-1-phosphate isomerase	256	*B. abortus*	Q2YIQ6
D-Erythrulose 1-phosphate 3-epimerase	315	*B. abortus*	Q2YIQ3
D-Erythritol 1-phosphate dehydrogenase	510	*B. abortus*	Q2YIQ2
Erythritol kinase	520	*B. abortus*	Q2YIQ1
Erythritol-4-phosphate dehydrogenase	502	*Brucella melitensis*	AAL53671.1
Erythritol/L-threitol dehydrogenase	368	*Mycolicibacterium smegmatis*	A0QXD8
Erythritol-4-phosphate dehydrogenase	503	*Sinorhizobium fredii* (syn. *Ensifer fredii*)	AAQ87123.1

^*a*^ Last accessed 3 March 2024.

Further, the genomes of *L. fermentum* IMDO 130101 and IMDO TC9L10 were compared against each other to assess their degree of similarity. To do so, the DNAdiff tool from MUMmer3 was used to determine the genome lengths, average nucleotide identity (ANI) values, and genomic differences due to insertions or deletions (39).

### Starter culture-initiated wheat sourdough productions

Sourdough productions were carried out with commercial wheat flour (provided by Puratos, Groot-Bijgaarden, Belgium). On a dry matter basis (m/m), this wheat flour had the following characteristics: moisture content, 9.7% (40); Hagberg falling number, 357 s (Perkin-Elmer, Waltham, Massachusetts, USA); damaged starch, 6.6% (41; SDMatic, Chopin, Villeneuve-la-Garenne, France); dry gluten content, 9.5% (Glutomatic 2000, Perkin-Elmer); and gluten index, 99.5% (Glutomatic 2000, Perkin-Elmer). The flour was stored in a humidity-controlled storage unit (N’ice holding cabinet; Irinox, Conegliano, Italy) at 7°C–10°C until use.

Starter culture-initiated wheat sourdough productions were performed in duplicate to obtain sourdoughs of 1.0 kg with a dough yield of 200, using either *L. fermentum* IMDO 130101 or IMDO TC9L10. Briefly, equal amounts of flour and water were mixed with a hand mixer (Bosch, Stuttgart, Germany), after which the mixtures were inoculated (1.0%, v/m). The inoculum build-up described above was carried out in mMRS-5 medium. Sampling was done at time 0, 0’ (after inoculation), 4, 8, 12, 24, and 48 h, to determine the pH, total titratable acidity (TTA), microbial counts, culture-independent microbial community dynamics, and metabolite dynamics, as described below (Sections 4.5, 4.6, and 4.7).

### Fermentation processes in wheat sourdough simulation medium

#### Small-scale fermentation processes

The small-scale fermentation processes were carried out in 100 mL glass bottles with *L. fermentum* IMDO 130101. The bottles were pre-filled with 100 mL of WSSM and sterilized at 121°C for 20 min. After the above-described inoculum build-up in WSSM, the inoculation was done at 1.0% (v/v). The fermentation temperature was 30°C. Sampling was done at time 0, 0’ (after inoculation), 3, 6, 12, 24, and 36 h, to determine the pH, microbial counts, and metabolite dynamics, as described below (Sections 4.5, 4.6, and 4.7). These fermentation processes were carried out in duplicate.

#### Medium-scale fermentation processes

The medium-scale fermentation processes were carried out in 2 L Biostat BDCU fermentors (Sartorius, Melsungen, Germany) with *L. fermentum* IMDO 130101. The fermentors were filled with 2 L of WSSM and sterilized at 121°C for 20 min, excluding the carbohydrates (maltose, sucrose, glucose, and fructose), which were added to the fermentors after a separate sterilization step of the aqueous solutions, prior to inoculation. After the above-described inoculum build-up in WSSM, the inoculation was done at 1.0% (v/v). The temperature (30°C) and agitation (100 rpm) were controlled online. Aeration was not provided. The production of carbon dioxide during fermentation was followed online by means of a Compact GC (cGC), as described previously (42). A Loess regression was used to model the carbon dioxide concentration data, followed by a k-fold cross-validation with the caret package (42). Sampling was done at time 0’ (after inoculation), 3, 6, 9, 12, 15, 24, 36, and 48 h, to determine the pH, TTA, optical density, biomass, microbial counts, and metabolite dynamics, as described below (Sections 4.5, 4.6, and 4.7). These fermentation processes were carried out in triplicate.

### Physicochemical analyses

Determinations of the pH and TTA values, the latter expressed as the number of ml of 0.1 N NaOH, were carried out as described previously (22).

### Microbiological analyses

#### Microbial growth

Microbial growth was followed by OD_600_ and biomass (gravimetrically, as cell dry mass) determinations, as described previously (42). Briefly, the OD_600_ values were determined with a spectrophotometer (Genesys 20; Sigma-Aldrich); ultrapure water (MilliQ; EMD Millipore, Burlington, Massachusetts, USA) was used as a blank. The cell dry mass values were determined by membrane filtration of the sample, using a cellulose nitrate filter (VWR International), after which the retentate was oven-dried at 105°C for 24 h.

#### Selective plating and microbial counts

To determine the viable counts of presumptive LAB and yeasts, appropriate decimal dilutions of the samples were prepared in saline, and plated on selective agar media, using mMRS-5 and yeast-peptone-glucose (YPG) agar media, respectively, as described previously (22).

#### Culture-independent microbial community dynamics

Cell pellets obtained through centrifugation (4,600×*g*, 20 min, 4°C) of the samples were subjected to total DNA extraction, as outlined previously (22). Amplicon-based high-throughput sequencing was applied to follow the whole-community microbial diversity dynamics. Two different sequencing strategies were used for the bacterial and fungal communities. The bacterial DNA was processed as described previously (43). Briefly, the full-length 16S rRNA gene was amplified using the primer set 27F and 1492R (Integrated DNA Technologies, Leuven, Belgium). The primers were tagged with 5’ sample-specific barcodes to allow for multiplex sequencing. The PCR assays were performed using KAPA HiFi DNA Polymerase (Roche, Basel, Switzerland). The post-amplification quality control was done with a Bioanalyzer 2100 and a High Sensitivity DNA Kit (Agilent Technologies, Santa Clara, California, USA). The DNA concentrations were measured with a Qubit 2.0 fluorometer (Thermo Fisher Scientific, Waltham, Massachusetts, USA). The amplicon sets were pooled equimolarly, and a circular sequencing adapter was ligated. Sequencing was performed using a PacBio Sequel system (PacBio, Menlo Park, California, USA) in circular consensus mode (VIB Nucleomics Core Facility, Leuven, Belgium). Circular consensus sequences (CCS) were generated using SMRT Link (PacBio), with a minimum predicted accuracy of 0.998. The internal transcribed spacer (ITS) region (ITS1) of the fungal rRNA transcribed unit was amplified using the primers BITS and B58S3 (44), as described previously (22). The microbial community dynamics was expressed as relative abundances based on the percentage of the total number of sequence reads obtained.

To infer the ASVs, both the bacterial and fungal sequences were processed with the R package DADA2 (42), following a pipeline described previously (45, 46). For the 16S rRNA amplicons, the following filtering parameters were applied: minQ = 3, minLen = 1100, maxLen = 1600, maxN = 0, rm.phix = FALSE, and maxEE = 2. For the ITS1 region amplicons, the parameters maxN = 1, truncQ = 2, maxEE = 2, minLen = 50, truncLen = 200, and rm.phix = TRUE were applied. Taxonomy was assigned with the SILVA database (version 138; ; 47) for the bacterial ASVs, and with the UNITE database (https://unite.ut.ee; 29.11.2022; 48) for the fungal ASVs. Only taxa with relative abundances above 0.5% in at least one of the samples were reported. The others were pooled together as “Minorities.” When ASVs were given identical taxonomic assignments, their relative abundances were summed. In a second instance, the ASVs belonging to the Lactobacillales order were selected and the relative abundances of these different ASVs for each genus were reported.

### Metabolite target analysis

To map the substrate consumption and metabolite production dynamics, the sample supernatants of all fermentation processes were used to perform a dedicated metabolite target analysis, as described below. All measurements were performed in triplicate. External standards of pure compounds, purchased from Sigma-Aldrich, were used for quantification.

#### Determination of monosaccharides, disaccharides, and sugar alcohols

The concentrations of monosaccharides (arabinose, fructose, galactose, glucose, mannose, and xylose) and disaccharides (maltose and sucrose) were determined by high-performance anion exchange chromatography with pulsed amperometric detection (HPAEC-PAD), as described previously (23), using an ICS 6000 chromatograph equipped with a CarboPac PA20 column in combination with an ED-40 PAD detector (Dionex; Thermo Fisher Scientific, Sunnyvale, California, USA).

The concentrations of sugar alcohols (erythritol, glycerol, mannitol, and sorbitol) were determined in a similar way, using an ICS 6000 chromatograph equipped with a CarboPac MA1 column (Dionex), as detailed previously (22).

#### Determination of organic acids

The concentrations of organic acids (citric acid, fumaric acid, gluconic acid, glucuronic acid, lactic acid, malic acid, and succinic acid) were determined by ultrahigh-performance liquid chromatography with tandem mass spectrometry detection (UPLC-MS/MS), using an Acquity UPLC system (Waters, Milford, Massachusetts, USA), equipped with a HSS T3 column and a triple-quadrupole (TQ) tandem mass spectrometer (Waters), as described previously (22).

The ratio of D-lactic acid to L-lactic acid was determined by UPLC-MS/MS with an Acquity UPLC system (Waters), equipped with an Astec Chirobiotic T column v04 (Supelco, Bellefonte, Pennsylvania, USA) and coupled to a TQ tandem mass spectrometer (Waters), as described previously (43).

#### Determination of ethanol, short-chain fatty acids, and low-molecular-mass volatile organic compounds

The concentrations of ethanol, short-chain fatty acids (acetic acid, propionic acid, butyric acid, isobutyric acid, valeric acid, isovaleric acid, and hexanoic acid), and low-molecular-mass volatile organic compounds (3-methyl-1-butanol, acetoin, 2-butanone, diacetyl, ethyl acetate, ethyl lactate, isoamyl acetate, and furfural) were determined by gas chromatography with flame ionization detection (GC-FID), using a Trace Finder 1310 gas chromatograph equipped with a DBwax-UI column (Thermo Fisher Scientific) and a FID-80 detector (Interscience, Breda, The Netherlands), as described previously (22).

### Statistical analysis

The data processing and statistical analyses were conducted in RStudio (49).

The carbon recovery was calculated for the WSSM fermentation processes as the total carbon equivalents of metabolites produced, encompassing lactic acid, acetic acid, ethanol, carbon dioxide (only for the medium-scale fermentation experiments), succinic acid, mannitol, glycerol, and erythritol, divided by the total carbon equivalents of substrates consumed, encompassing maltose, sucrose, glucose, fructose, and malic acid. The values used were those based on the chromatography determinations of these substrates and metabolites.
